# Synaptic protein levels altered in vascular dementia

**DOI:** 10.1111/nan.12215

**Published:** 2015-04-23

**Authors:** Lindsey I Sinclair, Hannah M Tayler, Seth Love

**Affiliations:** * School of Social and Community Medicine, University of BristolBristol, UK; † School of Clinical Sciences, University of BristolBristol, UK

**Keywords:** apolipoproteins E, DLG4 protein human, drebrins, SNAP 25 protein human, synaptophysin, vascular dementia

## Abstract

**Introduction:**

Cerebral ischaemia is the defining pathophysiological abnormality in most forms of vascular dementia (VAD), but the pathogenesis of the dementia remains poorly understood. In Alzheimer's disease (AD), there is early loss of synaptic proteins, but these have been little studied in VAD.

**Materials and Methods:**

We measured synaptophysin, postsynaptic density protein 95 (PSD-95), drebrin, synaptosomal-associated protein 25 (SNAP-25) and vascular endothelial growth factor (VEGF) by enzyme-linked immunosorbent assays in superior temporal cortex from 11 patients with VAD and, initially, 11 non-dementia controls. We corrected for neuronal content by measurement of neuron-specific enolase. A further 11 controls were subsequently used in a validation study. Simulation of post-mortem delay found that PSD-95 was stable at 4°C but declined slightly at RT. SNAP-25 and drebrin showed good post-mortem stability. Previous studies had shown good post-mortem preservation of synaptophysin and VEGF.

**Results:**

The VAD cases had lower synaptophysin (but *P* > 0.05 in initial study), significantly lower SNAP-25 (*P* = 0.024) and significantly higher drebrin (*P* = 0.020). On comparison with the second control group, the reduction in synaptophysin was significant (*P* = 0.008), and the other results were confirmed.

**Conclusion:**

There is probably a reduction in presynaptic proteins in the temporal cortex in VAD, although not as marked as in AD. In VAD, there is also an increase in drebrin, which may be a response to reduced synaptic input.

## Introduction

Although it is a major cause of cognitive decline, with an age-adjusted incidence estimated at 11–13 per 1000 per year 1–3, vascular dementia (VAD) has been studied biochemically to a much lesser extent than have other types of dementia. This is partly because it has proven difficult to define in research terms [4],[5]. Ongoing Delphi consensus studies (VICCCS and VCIN) aim to produce a single set of clinical and neuropathological criteria which can be used in research [6]. Nevertheless, clinicians are able to use the ICD-10 or DSM criteria [7],[8] to make a clinical diagnosis of dementia which is considered to result from ischaemic damage − sometimes acute and widespread (e.g. poststroke dementia) but more usually the cumulative effect of multiple, smaller, temporally and topographically dispersed ischaemic events [5],[9]. Clinically, a distinction is sometimes drawn between the gradual cognitive decline of Alzheimer's disease (AD) and the stepwise disease progression of VAD, each ‘step’ presumed to be a result of a separate infarct. In VAD, episodic memory impairment is often initially less prominent than in AD, with attention and executive function being more severely impaired [9].

In recent years, it has become increasingly recognized that while VAD and AD can be separate disease states, there are many patients in whom the two coexist. The two diseases also share several risk factors [1],[3],[10]. There is evidence that reduced cerebral blood flow may contribute to the progression of AD 11–15 and conversely that the accumulation of Aβ in AD not only contributes directly to small vessel disease (SVD) through the development of cerebral amyloid angiopathy but also reduces blood flow by enhancing vasoconstriction 16–20.

Synaptic loss was recognized nearly 30 years ago as a major neuropathological abnormality in AD [21]. It is thought that this loss reflects a combination of neuronal loss and synaptic degeneration. In AD, this process is predominantly presynaptic [22]. A recent proteomics study which looked at the synaptosome in AD *vs.* controls found differences in expression of a wide range of proteins involved in processes such as vesicular trafficking, synapse structure and signal transduction [23].

In contrast to AD, synaptic proteins have been little studied in VAD. Two very small previous studies of forms of VAD found a similar reduction in synaptophysin to that in AD [24],[25]. SNAP-25, PSD-95 and drebrin have not previously been studied in VAD. We have addressed this by measuring concentrations of synaptophysin, SNAP-25, PSD-95 and drebrin in post-mortem superior temporal cortex from patients with VAD and nondemented controls. The proteins under investigation were chosen to provide information on both pre and postsynaptic integrity.

Synaptophysin is a 313-amino acid, 38-kDa presynaptic vesicle-specific protein [26],[27] which can be used as a marker of synaptic content. It is not specific to any type of neuron [27]. Synaptophysin is significantly reduced in AD, with some evidence to suggest that it is affected by *APOE* genotype 28–31. Synaptophysin concentration has been shown to correlate with the level of cognitive impairment in AD 32–34. A reduction was also demonstrated in post-mortem brain from individuals with Braak tangle stages of III and above but no clinical dementia prior to death [33]. Synaptosomal-associated protein 25 (SNAP-25) is part of the SNARE complex involved in synaptic vesicle membrane docking and fusion. It was shown to be significantly decreased in AD brains and in frontotemporal dementia 26,28,35–37.

Postsynaptic density protein 95 (PSD-95) is a 724-amino-acid postsynaptic protein [26]. It is a member of the membrane-associated guanylate cyclase family [38], interacts with glutamate receptors (NMDA type) and is required for the synaptic plasticity associated with these receptors [26]. It is also located in dendritic spines and is important for spine stability [39]. PSD-95 was found to be reduced significantly in AD and minimal cognitive impairment (MCI, considered to be a possible AD prodrome) [29],[40],[41]. Drebrin, a 649-amino acid protein, subject to post-translational modification [26] and involved in dendritic spine morphogenesis, is also significantly reduced in AD and MCI 31,42–44.

Vascular endothelial growth factor A (VEGF) is a cytokine that is predominantly expressed by astrocytes in the human brain and plays a pivotal role in hypoxia-induced angiogenesis [45]. It was previously reported that only trace levels are found in healthy human brain, although it is known to be produced constitutively and may play a role in hippocampal neurogenesis 45–47. The CSF level of VEGF was found to be raised in AD and VAD [46].

We hypothesized that synaptic proteins which have been shown to be reduced in post-mortem brain samples in AD would also be reduced in VAD, with a reduction in synaptophysin as our primary outcome measure. We chose to study tissue from the superior temporal gyrus as this region of cortex has reduced synaptic proteins in AD, and infarcts have been shown to be present in the temporal lobe in >90% of patients with VAD [48].

## Materials and methods

### Case selection

Brain tissue was obtained from 11 VAD cases and, initially, 11 controls, as shown in Table 1. Further information is available in supplementary table 1. Tissue was supplied by the South West Dementia Brain Bank (SWDBB), University of Bristol; the Sudden Death Brain and Tissue Bank (SDBTB), University of Edinburgh; and the London Neurodegenerative Diseases Tissue Bank, King's College London (LNDTB). The cases had a mean post-mortem delay which was almost double that of the controls (64.5 h *vs.* 34.5 h, *P* = 0.007). All work was approved under tissue bank generic ethical approval for peer-reviewed projects.

**Table 1:** Individual-level characteristics of the samples included in this study

ID	Case/control	Sex	Age at death	PMD (h)	Braak stage	Clinicopathological diagnosis	SVD score (temporal)
1	Case	M	81	66	0	VAD	3
2	Case	M	83	24	II	VAD	2
3	Case	F	80	70	II	VAD	0
4	Case	M	72	41	III	VAD	2
5	Case	F	77	85	I	VAD	0
6	Case	F	79	88	II	VAD	3
7	Case	M	67	54	III	VAD	2
8	Case	M	76	40	III	VAD	3
9	Case	M	79	56	II	VAD	1
10	Case	M	79	77	I	VAD	3
11	Case	M	82	109	N/A	VAD, DLBD	2
12	Control	M	73	51	0	Asymptomatic small vessel disease	N/A
13	Control	M	74	44	0	Normal	N/A
14	Control	M	75	47	II	Small vessel disease	N/A
15	Control	F	72	30	III	Small vessel disease	N/A
16	Control	M	73	23	0	Normal	N/A
17	Control	M	74	22.5	I	Amyloid angiopathy	N/A
18	Control	M	75	48	II	Normal	1
19	Control	M	75	6	III	Normal	N/A
20	Control	M	73	35	III	Normal	0
21	Control	F	74	39.5	I	Normal	1
22	Control	M	73	33	I	Normal	1
23	Control (2)	M	82	3	II	Normal	1
24	Control (2)	F	78	24	II	Normal	1
25	Control (2)	M	69	66	II	Normal	1
26	Control (2)	M	72	42	I	Normal	0
27	Control (2)	M	79	24	N/A	Normal	N/A
28	Control (2)	F	76	106	II	Normal	1
29	Control (2)	F	76	12	N/A	Normal	N/A
30	Control (2)	M	78	48	II	Normal	1
31	Control (2)	M	76	23	II	Normal	1
32	Control (2)	M	78	12	II	Normal	0
33	Control (2)	M	81	3	II	Normal	0

Small vessel disease (SVD) scores were not available for samples which were provided by the Sudden Death Brain and Tissue Bank and the London Neurodegenerative Diseases Tissue Bank. F, female; M, male; N/A, not available; PMD, post-mortem delay; VAD, vascular dementia.

All cases had VAD which met DSM-IV criteria, a Braak tangle stage of III or less, no more than sparse neuritic plaques, neuropathological evidence of multiple infarcts or regions of ischaemic damage, moderate to severe atherosclerosis and/or arteriosclerosis, and an absence of histopathological evidence of other disease processes likely to cause dementia [49],[50]. The controls had no evidence of dementia at the time of their death and fulfilled most of the same neuropathological criteria as the cases apart from a lack of moderate or severe atherosclerosis or arteriosclerosis, detectable infarcts, or other ischaemic lesions. This work was part of a larger study examining synaptic markers in controls aged 75 and under; so, all controls were aged 75 or less. For this reason, cases and controls were not matched exactly for age in the first part of the study.

To validate our initial findings, a second control group was used which was much more closely matched with the VAD cases for age, as shown in Table 2. All of the second set of control samples were provided by the SWDBB. To ensure comparability between cases and controls in the validation assays, further aliquots of brain homogenate from the VAD cases were included on the same microplates as the second set of controls.

**Table 2:** Summary statistics for the samples from the second control group and cases included in this study

		Control group 2 mean (SD)	Case mean (SD)	*P* value (*t*-test, Wilcoxon rank sum or regression)
Gender	Male	8	8	
	Female	3	3	
Age at death		76.8 (3.74)	77.7 (4.67)	0.620
Post-mortem delay		33.0 (31.1)	64.5 (24.7)	0.012
Braak stage		1.78 (0.44)	1.90 (0.99)	0.739
Temporal SVD score		1.00 (0.00)	1.57 (1.27)	
Synaptophysin (ng/μl)		3.95 (2.21)	1.62 (0.63)	0.008
Drebrin (ng/μl)		3.87 (1.47)	5.53 (1.13)	0.012
SNAP-25 (ng/μl)		0.38 (0.22)	0.017 (0.003)	<0.001

As can be seen, the cases have a significantly greater post-mortem delay. For this comparison, adjustments were used for the average NSE and total protein measurements for these two groups, which accounts for the difference in the case values between this comparison and that with the first control group.

### Brain tissue

Tissue from the superior temporal gyrus (Brodmann area 21/22) was dissected from brains that had been removed from patients as soon as practicably possible after death and then frozen at −80°C. In all bar 5 instances, this was within 72 h of death. The tissue provided by the SDBTB and LNDTB had already been dissected. For each sample, 200 mg of tissue was homogenized in 1 ml chilled 1% SDS lysis buffer in a Precellys homogenizer (2 × 15 s at 6000 **g**) with 6–10 zirconia beads in a 2-ml screw cap homogenate tube. The homogenates were then centrifuged at 13 000 **g** for 15 min at 4°C. The supernatants were kept on ice and aliquoted into 25-μl aliquots and stored at −80°C until required for analysis.

SVD scoring of paraffin-fixed temporal sections had already been performed on a 4-point scale, as described in detail by Barker *et al*. [51], with 0 representing normal vessel wall thickness, 1 slightly increased wall thickness, 2 moderately increased thickness and 3 markedly increased thickness such that for many arterioles, the diameter of the lumen was <50% of the outer diameter of the blood vessel.

### Measurement of synaptic proteins

Drebrin, synaptophysin and VEGF were measured by sandwich enzyme-linked immunosorbent assays (sandwich ELISA). SNAP-25 and PSD-95 were measured by direct ELISA assays. Details of the antibodies used are shown in Table 3. All measurements were performed at least in duplicate, and the mean values were used in the subsequent analyses. All measurements were corrected for protein concentration and neuron-specific enolase concentration to account for differences in neuronal content, as described in detail by Ashby *et al*. [52] and Miners *et al*. [20].

**Table 3:** Antibodies used in the ELISAs in this study

Target	Clonality	Species raised in	Company and product code	Epitope
Synaptophysin	Polyclonal	Rabbit	Abcam ab53166	Proprietary
Synaptophysin	Monoclonal	Mouse	Santa-Cruz Biotech sc17750	aa 221–313 of human synaptophysin
Neurone-specific enolase	Polyclonal	Rabbit	Biomol BML-NA1247	aa 269–286 of human NSE
Neurone-specific enolase	Monoclonal	Mouse	Abcam ab24472	Proprietary
Drebrin	Monoclonal	Mouse	Abcam ab60932	Proprietary
Drebrin	Polyclonal	Rabbit	Abcam ab11068	Proprietary
SNAP-25	Monoclonal	Mouse	Santa-Cruz Biotech sc376713	aa 91–140 of human SNAP-25
PSD-95	Monoclonal	Mouse	Sigma-Aldrich P246	Proprietary

### NSE

The rabbit polyclonal capture antibody (Enzo Life Sciences, Farmingdale, NY, USA) was diluted 1:1000 in 1× coating buffer and incubated at 4°C overnight in clear 96-well microplates. Following five washes with 0.05% PBS/Tween 20 (PBST), the plate was tapped dry, and non-specific protein binding was blocked by incubation with 1% BSA/PBS (Bovine serum album in phosphate buffered saline) at room temperature (RT) for 1 h with agitation. Following a further five washes in PBST, the plate was tapped dry, and five serial dilutions of recombinant NSE (Abcam, Cambridge, UK) in PBS, blanks consisting of PBS, and homogenates diluted 1:10 in PBS were all loaded in duplicate. The plate was incubated for 2 h at RT with agitation. After five washes in PBST, peroxidase-labelled mouse monoclonal detection antibody (Abcam), diluted 1:1000 in PBS, was added and incubated for 2 h in the dark at RT, with agitation. After a final five PBST washes, the peroxidase substrate (100 μl per well) was applied for 10 min, at the end of which 50 μl of STOP solution was added. Absorbance at 450 nm was read in a multidetection microplate reader (Fluostar OPTIMA, BMG Labtech, Aylesbury, UK). Absolute protein levels were interpolated from the standard curve.

### Drebrin

The rabbit polyclonal capture antibody (Abcam) was diluted 1:3000 in 1× coating buffer and incubated at 4°C overnight in clear 96-well microplates. Following five washes with 0.05% PBST, the plate was tapped dry, and non-specific protein binding was blocked by incubation with 1% BSA/PBS at RT for 1 h with agitation. Following a further five washes in PBST, seven serial dilutions of the recombinant drebrin (Abnova, Taipei City, Taiwan) in PBS, blanks consisting of PBS and homogenates diluted 1:100 in PBS were loaded in duplicate. The concentration of the protein standard ranged from 0.8 to 0.0125 ng/μl. The plate was incubated for 1 h at RT with agitation. After a further five washes in PBST, mouse monoclonal detection antibody (Abcam) diluted 1:3000 in 1% BSA/PBS was added for 1 h at RT, with agitation. After a further five washes, the plate was incubated with peroxidase-labelled antimouse secondary antibody (Vector Laboratories, Burlingame, CA, USA) diluted 1:500 in PBS for 30 min. After a final five washes with PBST, 100 μl of peroxidase substrate (R&D systems, Minneapolis, MN, USA) was added to each well, and the plate allowed to develop for 5 min, at the end of which 50 μl of STOP solution was added. Absorbance at 450 nm was read in a multidetection microplate reader. Absolute protein levels were interpolated from the standard curve.

### Synaptophysin

The rabbit polyclonal capture antibody (Abcam) was diluted 1:1000 in 1× coating buffer and incubated at 4°C overnight in clear 96-well microplates. Following five washes with 0.05% PBST, the plate was tapped dry, and non-specific protein binding was blocked by incubation with 1% BSA/PBS at RT for 1 h with agitation. Following a further five washes in PBST and the plate being tapped dry, five serial dilutions of the recombinant protein standard (Abnova) in PBS, blanks consisting of PBS and homogenates diluted in PBS such that 10 μg of total protein was loaded per well, were loaded in duplicate. The concentration of the protein standard ranged from 5 to 0.313 μg/μl. The plate was then incubated for 1 h at RT with agitation. After a further five washes in PBST, the plate was tapped dry, and mouse monoclonal detection antibody (SantaCruz Biotechnology, Dallas, TX, USA) diluted 1:1000 in 1% BSA/PBS was added for 1 h at RT, with agitation. After a further five washes, the plate was incubated with biotinylated antimouse secondary antibody diluted 1:500 in PBS (Vector Laboratories) for 20 min. After a further five washes, the plate was incubated with streptavidin horse radish peroxidase (R&D systems) diluted 1:500 in PBS for 20 min. After a final 5 washes with PBST and the plate being tapped dry, 100 μl of peroxidase substrate was added to each well and the plate allowed to develop for 10 min, at the end of which 50 μl of STOP solution was added. Absorbance at 450 nm was read using a multidetection microplate reader. Absolute protein levels were interpolated from the standard curve.

### VEGF

VEGF was assayed by means of a commercially available sandwich ELISA kit (R&D systems), according to the manufacturer's instructions, as previously [53].

### PSD-95

Brain homogenates diluted 1:20 in PBS and blanks of PBS alone were incubated for 2 h at 26°C in a clear 96-well microplate (Fisher Scientific, Loughborough, UK). The plate also included seven threefold serial dilutions of recombinant PSD-95 (Abnova) over a concentration range of 0.90 ng/μl to 0.0012 ng/μl. The plate was washed five times in 0.05% PBST, tapped dry and then incubated for 2 h at 26°C with mouse monoclonal antibody to PSD-95 (Sigma Aldrich, St Louis, MO, USA) diluted 1:3000 in 1% BSA/PBS. After a further five washes, the plate was incubated with peroxidase-labelled anti-mouse secondary antibody (Vector Laboratories) diluted 1:500 in PBS for 30 min. After a final five washes, 100 μl of peroxidase substrate was added to each well, and the plate was allowed to develop for 10 min, at which point 50 μl of STOP solution was added to each well. Absorbance at 450 nm was read in a multidetection microplate reader. Absolute protein levels were interpolated from the standard curve.

### SNAP-25

Brain homogenates diluted 1:6.7 in 1× coating buffer and blanks of 1× coating buffer alone were incubated for 2 h at 26°C in a clear 96-well microplate. The plate also included seven threefold serial dilutions of recombinant SNAP-25 (Abcam) over a concentration ranging from 2.0 to 0.02 ng/μl. The plate was washed five times in 0.05% PBST, tapped dry and then incubated for 2 h at 26°C with the mouse monoclonal antibody to SNAP-25 (SantaCruz Biotechnology) which was diluted 1:3000 in 1% BSA/PBS. After a further five washes, the plate was incubated with peroxidase-labelled anti-mouse secondary antibody diluted 1:500 in PBS (Vector Laboratories) for 30 min. After a final five washes, 100 μl of peroxidase substrate was added to each well, and the plate allowed to develop for 10 m, at which point 50 μl of STOP solution was added to each well. Absorbance at 450 nm was read using a multidetection microplate reader. Absolute protein levels were interpolated from the standard curve.

### Measurement of post-mortem stability of synaptic proteins

NSE, synaptophysin, drebrin and VEGF concentrations had previously been shown not to be affected by post-mortem delay up to 72 h [29],[52],[53]. PSD-95 and drebrin had been studied in a previous, unpublished study from our group (Figure S2). On incubation of multiple aliquots of cerebral cortex from three brains for periods of up to 72 h at RT or 4°C, PSD-95 concentration showed a small but significant decrease over 72 h at RT (Spearman's *r* −0.8929, *P* = 0.0123) but no significant decrease at 4°C. Drebrin had been measured in this previous study on the same set of homogenates by scanning densitometry of Western blots, the method for which has been published previously [54]. There was no significant change in drebrin concentration at either RT or 4°C.

Post-mortem delay was also simulated for SNAP-25 by taking multiple samples of tissue from the temporal lobe of four brains with a short post-mortem delay and incubating them for 6, 12, 18, 24, 48 and 72 h at RT and 24, 48 and 72 h at 4°C. The tissue was then homogenized, and the homogenates were stored at −80°C prior to protein measurements being made as described above.

### Statistical analysis

The power calculation for this study was based on the finding of Love *et al*. [29] that synaptophysin, as measured by sandwich ELISA in the superior temporal cortex, was reduced by approximately 2/3 in AD compared with controls. The present study was powered to find a similar or slightly smaller difference in VAD, with synaptophysin level as the primary outcome measure. A sample size of 11 per group gave 82% power to find a difference 80% of that found by Love *et al*. [29].

Where possible, parametric statistical tests were used. If variables were not normally distributed, log logarithmic transformations were used to obtain a normal distribution. For normally distributed variables, the *t*-test was used. For variables that were not normally distributed, even after logarithmic transformation, the Wilcoxon rank sum test was used. Where post-mortem delay had been shown to affect protein levels, we used linear regression with post-mortem delay included as a variable. If the residuals were not normally distributed (and thus the assumptions of linear regression violated), then the variable was log transformed and the distribution of the residuals rechecked. A threshold for *P* values of 0.05 was used throughout. Spearman's correlation was used to assess the effect of post-mortem delay.

## Results

The first part of the study comprised 11 controls and 11 VAD cases. In the second part of the study, we used a further 11 controls. The individual characteristics of each sample are summarized in Table 1. The comparison between the VAD cases and the original control group is summarized in Table 4 and that with the second control group in Table 2. The post-mortem delay was significantly greater for the VAD cases (*P* = 0.007). The cases were slightly older than the first set of controls (mean difference = 4 yrs, *P* = 0.012) but were well matched for gender. The second control group were well matched for both age (mean difference = 11 months, *P* = 0.620) and gender.

**Table 4:** Summary statistics for the samples included in this study

		Control mean (SD)	Case mean (SD)	*P* value (*t*-test, Wilcoxon rank sum or regression)
Gender	Male	9	8	
	Female	2	3	
Age at death		73.7 (1.01)	77.7 (4.67)	0.012
Post-mortem delay		34.5 (13.6)	64.5 (24.7)	0.007
Braak stage		1.45 (1.21)	1.90 (0.99)	0.382
Temporal SVD score		0.75 (0.50)	1.57 (1.27)	
Synaptophysin (ng/μl)		3.85 (2.68)	2.38 (0.75)	0.622
Drebrin (ng/μl)		5.47 (3.46)	8.38 (1.98)	0.020
SNAP-25 (ng/μl)		0.0373 (0.166)	0.0236 (0.0045)	0.024
PSD-95 (ng/μl)		0.411 (0.334)	0.480 (0.345)	0.876
VEGF (μg/μl)		0.573 (0.328)	0.830 (0.267)	0.058

As can be seen, the cases were older than the controls and had a greater mean post-mortem delay.

### Post-mortem stability

The concentration of SNAP-25 did not fall significantly with increasing time at RT (ρ = −0.2143, *P* = 0.6615) or at 4°C (ρ = −1.00, *P* = 0.0833), as shown in Figure S1.

### Presynaptic proteins

As can be seen in Table 4, the mean concentration of synaptophysin was higher in controls than in cases but not significantly so (38.2% difference, *P* = 0.622). When the analysis was repeated with the second group of controls, there was stronger evidence for a decrease in synaptophysin in VAD (59.0% difference, *P* = 0.008); note that this study was powered to detect an approximately 50% difference.

The mean concentration of SNAP-25 was higher in controls than cases, and there was strong evidence to support this from the Wilcoxon rank sum analysis (mean difference = 0.014 ng/μl, *P* = 0.024). The same direction of difference was observed in the new group of controls (mean difference = 0.363 ng/μl, *P* = < 0.001) suggesting that this is a true difference, rather than a type 1 error.

### Postsynaptic proteins

There was a very small increase in PSD-95 (0.7 ng/μl, *P* = 0.876) in the original comparison. Unfortunately, variability in the assay (as evidenced by comparison of values in the same VAD cases) made it impossible to obtain reliable data on comparing the new control group to the cases.

Drebrin was significantly increased in VAD. This was evident on comparison with the first set of controls as shown in Table 4. Drebrin concentration increased from a mean of 5.47 ng/μl in controls to 8.38 ng/μl in cases (*P* = 0.020). The findings were confirmed on comparison with the second control group as shown in Table 2, with a mean difference of 1.66 ng/μl (*P* = 0.012). This was an unexpected finding as drebrin is reduced in AD.

### Measures of vascular disease

Although temporal SVD scores were not available for all controls, the SVD score was higher in the cases (1.57 *vs*. 0.75). All of the controls for which data were available had a temporal SVD score of 1 or 0. There was a trend for higher VEGF levels in the cases (0.830 μg/μl *vs.* 0.573 μg/μl, *P* = 0.058). All of the cases had a neuropathological diagnosis of VAD. Several of the controls had some SVD documented at post-mortem examination, but we chose to include these to avoid selecting ‘super normal’ participants. Indeed, in one study >75% of nondemented older people had evidence of cerebrovascular disease at autopsy [55].

## Discussion

This study is the first to examine a range of synaptic proteins in VAD. All four of the synaptic proteins studied had previously been found to be decreased in AD compared with controls with no dementia at the time of death. The present study was powered with synaptophysin as the primary outcome and to find a similar or slightly lower decrease in synaptophysin to that in AD. We found a reduction in synaptophysin, nonsignificant in our original analysis, which may reflect a smaller reduction in VAD than in AD, but significant compared with our second group of controls.

The Cambridge later life study found in AD that in Braak tangle stages I and II, there was no change in synaptic proteins, including synaptophysin and SNAP-25, that levels increased in Braak stages III and IV and only decreased from Braak stage V onwards. The same pattern was seen in relation to clinical severity [56]. That was, however, a relatively small study. The authors suggested that the initial increase may represent an early adaptive synaptic regeneration which then fails as the disease process progresses. A similar study by Counts *et al*. [31] found no significant change in synaptophysin in five brain areas in MCI but a significant difference between controls and severe AD in the superior temporal and inferior parietal cortex. Our previous study which found a decrease in synaptophysin in AD analysed cases with a Braak stage of V or VI [29]. In the present study, the Braak tangle stages did not differ significantly between the cases and controls and would not be expected to contribute to any differences between the groups.

We found a reduction in SNAP-25, as expected. Although the variable itself was not normally distributed, necessitating nonparametric testing, linear regression was possible because the residuals were normally distributed.

Surprisingly, we found an increase in drebrin. This was contrary to our hypothesis that drebrin would be decreased, as in AD and MCI [29],[31],[44]. Drebrin has been shown to increase dendritic length, size and density [57] and be involved in NMDR receptor regulation [58]. Reduction of drebrin expression in cultured hippocampal neurons by use of antidense oligonucleotides led to decreased spine width and density [59],[60]. A study in rats following axotomy of spinal motor neurons found that drebrin expression increased in the lesioned motor neurons (compared with the unlesioned neurons) at 3 days and 7 days but had returned to normal after 10 weeks. The authors suggested that drebrin played a role in synaptic restoration [61]. Interestingly, a small study of transient cerebral ischaemia in rats found an increase in drebrin immunoreactivity in hippocampal area CA3 but not CA1, 7 days after ischaemia [62].

It seems most likely that the increase in drebrin seen in this study represents a compensatory response to the ischaemia caused by the VAD process. A microarray study of differing stages of AD *vs.* controls in post-mortem hippocampus found up-regulation of several processes including apoptosis, tumour suppressors, down-regulation of synaptic transmission and microtubule-based processes and a ‘collapse of protein transport machinery’ early in AD, although all of their categories of AD had an average Braak stage of V or more. This, in addition to the findings of the Cambridge Later Life Study, provides a precedent for proteins being up-regulated in dementia [56],[63].

Strengths of the present study include the range of measures of synaptic integrity, the repeated measurements of synaptic proteins in two independent control groups and the correction for neuronal content. Weaknesses include the low numbers, but this only became apparent *post hoc*: because these proteins had not previously been studied in VAD, figures from AD studies were used in the power calculations.

In summary, we have shown that although there is a reduction in presynaptic proteins (including a significant reduction in SNAP-25) in the temporal cortex in VAD, this is unlikely to be as large as that in AD. In VAD, there is also an unexpected increase in drebrin, which is probably a compensatory response to the disease process. However, further work is required, in larger cohorts, to confirm these findings.

## Supporting Information

**Figure S1.** The effect of post-mortem delay on SNAP-25. Post-mortem delay was simulated by the storage of aliquots of tissue at RT (A) or 4°C (B) for differing periods of time. Measurements were made on samples from more than one brain. The symbols are colour-coded (red, blue or black) to indicate which measurements were made on samples from each brain. The concentration of SNAP-25 did not fall significantly with increasing time at RT (ρ = −0.2143, *P* = 0.6615) or at 4°C (ρ = −1.00, *P* = 0.0833). **Figure S2.** The effect of post-mortem delay on PSD-95 and drebrin. Post-mortem delay was simulated by the storage of aliquots of tissue at RT or 4°C for differing periods of time. For the PSD-95 assays (**C** and **D**), measurements were made on samples from more than one brain. The symbols are colour-coded (red, blue or black) to indicate which measurements were made on samples from each brain. Post-mortem delay appeared to decrease drebrin concentration (**A** and **B**) slightly but not significantly. Spearman's ρ was −0.2413 at room temperature (*P* = 0.6615) and −0.2000 at 4°C (*P* = 0.9167). The concentration of PSD-95 was not significantly affected by storage at 4°C (**D**, Spearman's ρ −1.000, *P* = 0.0833) but did decline at room temperature (**C**, Spearman's ρ −0.8929, *P* = 0.0123). **Table S1** Individual level characteristics of the samples included in this study. Full post-mortem reports were not available for all samples.
