# Supplementary material for: Synaptic protein levels altered in vascular dementia
Source: Neuropathol Appl Neurobiol. 2015 Apr 23;41(4):533–43. doi: 10.1111/nan.12215 (PMC4471617; doi:10.1111/nan.12215)
Supplement: Supplementary file 1 — Figure S1. The effect of post-mortem delay on SNAP-25. Post-mortem delay was simulated by the storage of aliquots of tissue at RT (A) or 4°C (B) for differing periods of time. Measurements were made on samples from more than one brain. The symbols are colour-coded (red, blue or black) to indicate which measurements were made on samples from each brain. The concentration of SNAP-25 did not fall significantly with increasing time at RT (ρ = −0.2143, P = 0.6615) or at 4°C (ρ = −1.00, P = 0.0833). Figure S2. The effect of post-mortem delay on PSD-95 and drebrin. Post-mortem delay was simulated by the storage of aliquots of tissue at RT or 4°C for differing periods of time. For the PSD-95 assays (C and D), measurements were made on samples from more than one brain. The symbols are colour-coded (red, blue or black) to indicate which measurements were made on samples from each brain. Post-mortem delay appeared to decrease drebrin concentration (A and B) slightly but not significantly. Spearman's ρ was −0.2413 at room temperature (P = 0.6615) and −0.2000 at 4°C (P = 0.9167). The concentration of PSD-95 was not significantly affected by storage at 4°C (D, Spearman's ρ −1.000, P = 0.0833) but did decline at room temperature (C, Spearman's ρ −0.8929, P = 0.0123). Table S1 Individual level characteristics of the samples included in this study. Full post-mortem reports were not available for all samples. [file nan0041-0533-sd1.zip › NAN_12215_supp-0001-Supplementary Table 1 (SL 17-11-14).docx]

Supplementary Table 1: Individual level characteristics of the samples included in this study. Full post-mortem reports were not available for all samples

| **ID** | **Case/Control** | **White matter ischaemic changes^1^** | **Small vessel disease^1^** | **Multiple infarcts including micro-infarcts^1^** | **Summary of post-mortem neuropathology report^2^** |
| --- | --- | --- | --- | --- | --- |
| 1 | case | 1 | 1 | 1 |  |
| 2 | case | 1 | 2 | 1 | Numerous diffuse plaques especially temporal lobe, few NFTs in medial temporal cortex |
| 3 | case | 0 | 0 | 1 | No plaques, occasional NFTs |
| 4 | case | 1 | 1 | 1 | No plaques/NFTs |
| 5 | case | 1 | 1 | 1 | Very occasional NFT, no plaques |
| 6 | case | 0 | 2 | 2 | Moderate number diffuse plaques in temporal and occipital cortex, NFTs in medial temporal neocortex, moderate CAA |
| 7 | case | 2 | 2 | 1 | Occasional NFTs in entorrhinal cortex, nil elsewhere. No plaques |
| 8 | case | 2 | 2 | 1 | Few neuritic plaques. Moderate NFTs in entorrhinal cortex, few elsewhere |
| 9 | case | 1 | 2 | 0 | No evidence of Alzheimer disease |
| 10 | case | 2 | 2 | 1 | No Alzheimer changes |
| 11 | case | 1 | 2 | 1 | Occasional Lewy bodies especially medial temporal neocortex and cingulate gyrus |
| 12 | control | 0 | 0 | 1 | Asymptomatic small vessel disease |
| 13 | control | 0 | 0 | 0 | No significant abnormalities |
| 14 | control | 0 | 0 | 0 | Mild small vessel disease |
| 15 | control | 0 | 0 | 0 | Mild small vessel disease |
| 16 | control | 0 | 0 | 0 | No significant abnormalities |
| 17 | control | 0 | 1 | 0 | Amyloid angiopathy |
| 18 | control | 0 | 0 | 0 | Occasional NFT in hippocampus and entorrhinal cortex. No plaques. |
| 19 | control | n/a | n/a | n/a | n/a |
| 20 | control | 0 | 0 | 0 | Sparse diffuse plaques only |
| 21 | control | 0 | 0 | 0 | No significant abnormalities |
| 22 | control | 0 | 0 | 0 | No plaques or NFTs |
| 23 | control (2) | 0 | 1 | 0 | Scattered plaques in entorrhinal cortex and neocortex. Occasional plaques in hippocampus. |
| 24 | control (2) | 0 | 1 | 0 | No plaques seen. Occasional NFT in entorrhinal cortex, none in neocortex. |
| 25 | control (2) | 0 | 0 | 0 | No plaques or NFTs |
| 26 | control (2) |  |  |  | No plaques seen. Two NFTs entorrhinal cortex, nil in neocortex. |
| 27 | control (2) | 0 | 1 | 0 |  |
| 28 | control (2) | 0 | 0 | 0 | Moderate number of plaques in entorrhinal and medial temporal cortex. Fewer elsewhere. NFTs in hippocampus only. |
| 29 | control (2) | n/a | n/a | n/a |  |
| 30 | control (2) | 0 | 0 | 0 | Occasional NFTs in hippocampus and entorrhinal cortex only. No plaques |
| 31 | control (2) | 0 | 0 | 0 | Occasional NFTs in hippocampus and entorrhinal, none in neocortex cortex. No plaques |
| 32 | control (2) | 0 | 0 | 0 | No plaques. Occasional NFT in hippocampus only. |
| 33 | control (2) | 0 | 0 | 0 | Scattered NFTs in entorrhinal cortex, very few in temporal neocortex. None in frontal and parietal lobes. Scattered diffuse and neuritic plaques in temporal, frontal and parietal lobes. |

^1^ These were rated on a 0-2 scale where 0=mild/absent, 1=moderate, 2=severe

^2^ NFT= neurofibrillary tangle
