# Supplementary figures and images for: Synaptic protein levels altered in vascular dementia
Source: Neuropathol Appl Neurobiol. 2015 Apr 23;41(4):533–43. doi: 10.1111/nan.12215 (PMC4471617; doi:10.1111/nan.12215)

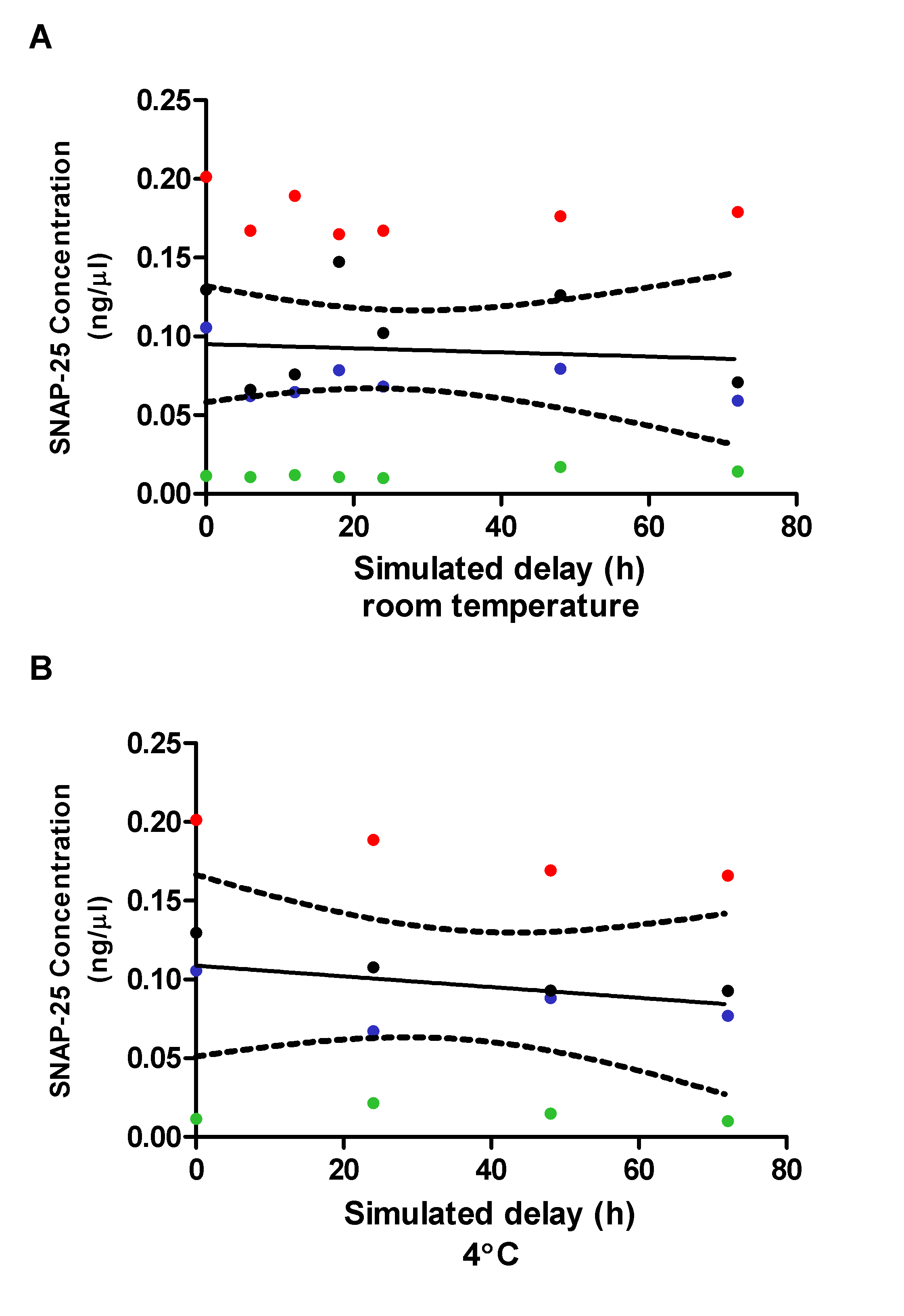

Supplement: Supplementary file 1 — Figure S1. The effect of post-mortem delay on SNAP-25. Post-mortem delay was simulated by the storage of aliquots of tissue at RT (A) or 4°C (B) for differing periods of time. Measurements were made on samples from more than one brain. The symbols are colour-coded (red, blue or black) to indicate which measurements were made on samples from each brain. The concentration of SNAP-25 did not fall significantly with increasing time at RT (ρ = −0.2143, P = 0.6615) or at 4°C (ρ = −1.00, P = 0.0833). Figure S2. The effect of post-mortem delay on PSD-95 and drebrin. Post-mortem delay was simulated by the storage of aliquots of tissue at RT or 4°C for differing periods of time. For the PSD-95 assays (C and D), measurements were made on samples from more than one brain. The symbols are colour-coded (red, blue or black) to indicate which measurements were made on samples from each brain. Post-mortem delay appeared to decrease drebrin concentration (A and B) slightly but not significantly. Spearman's ρ was −0.2413 at room temperature (P = 0.6615) and −0.2000 at 4°C (P = 0.9167). The concentration of PSD-95 was not significantly affected by storage at 4°C (D, Spearman's ρ −1.000, P = 0.0833) but did decline at room temperature (C, Spearman's ρ −0.8929, P = 0.0123). Table S1 Individual level characteristics of the samples included in this study. Full post-mortem reports were not available for all samples. [file nan0041-0533-sd1.zip › NAN_12215_supp-0002-Supplementary figure 1.tif]

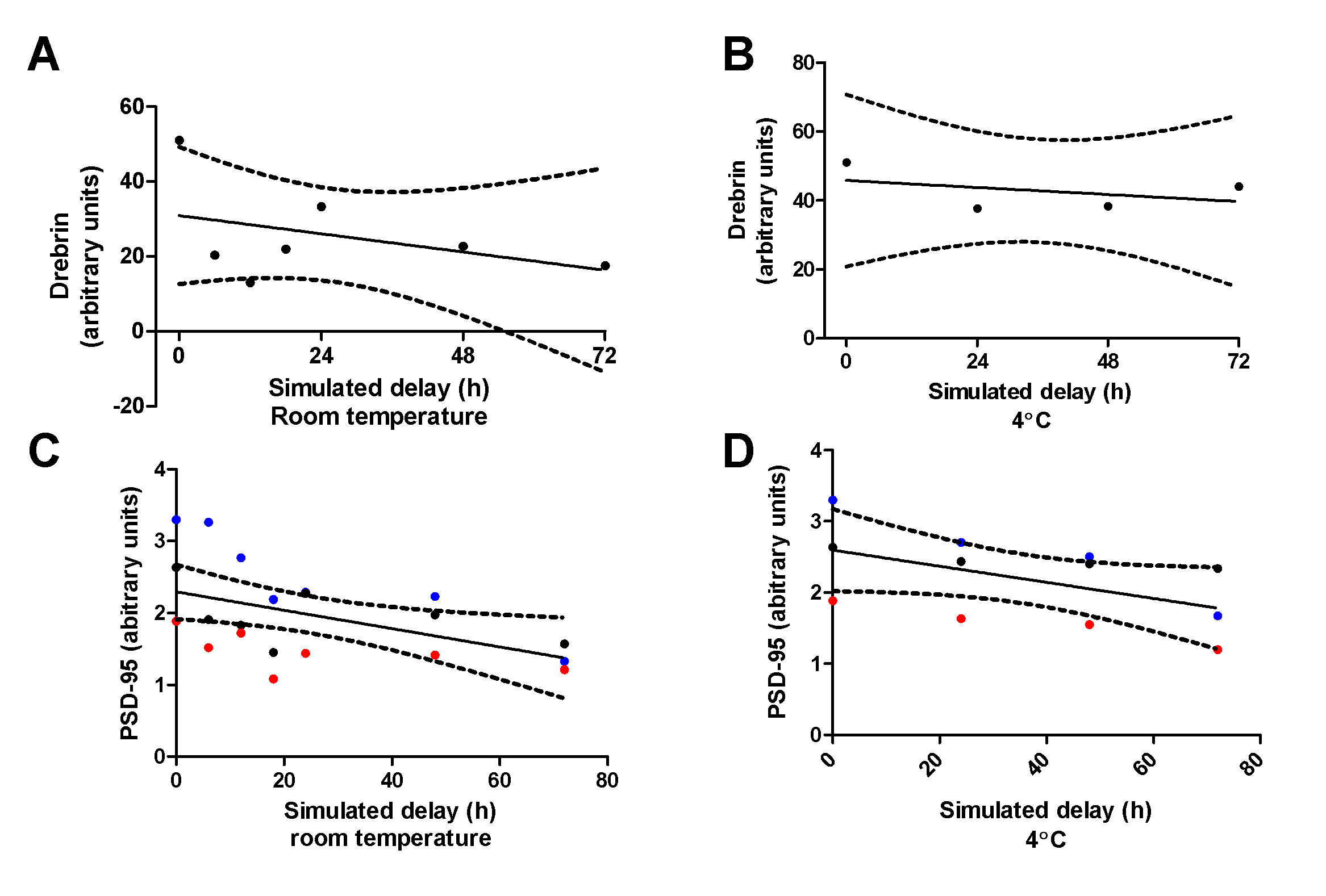

Supplement: Supplementary file 1 — Figure S1. The effect of post-mortem delay on SNAP-25. Post-mortem delay was simulated by the storage of aliquots of tissue at RT (A) or 4°C (B) for differing periods of time. Measurements were made on samples from more than one brain. The symbols are colour-coded (red, blue or black) to indicate which measurements were made on samples from each brain. The concentration of SNAP-25 did not fall significantly with increasing time at RT (ρ = −0.2143, P = 0.6615) or at 4°C (ρ = −1.00, P = 0.0833). Figure S2. The effect of post-mortem delay on PSD-95 and drebrin. Post-mortem delay was simulated by the storage of aliquots of tissue at RT or 4°C for differing periods of time. For the PSD-95 assays (C and D), measurements were made on samples from more than one brain. The symbols are colour-coded (red, blue or black) to indicate which measurements were made on samples from each brain. Post-mortem delay appeared to decrease drebrin concentration (A and B) slightly but not significantly. Spearman's ρ was −0.2413 at room temperature (P = 0.6615) and −0.2000 at 4°C (P = 0.9167). The concentration of PSD-95 was not significantly affected by storage at 4°C (D, Spearman's ρ −1.000, P = 0.0833) but did decline at room temperature (C, Spearman's ρ −0.8929, P = 0.0123). Table S1 Individual level characteristics of the samples included in this study. Full post-mortem reports were not available for all samples. [file nan0041-0533-sd1.zip › NAN_12215_supp-0003-supplementary figure 2.tif]
